# Acetazolamide for Bipolar Disorders: A Scoping Review

**DOI:** 10.3390/brainsci13010140

**Published:** 2023-01-13

**Authors:** Rebecca Strawbridge, Nefize Yalin, Stelios Orfanos, Allan H. Young

**Affiliations:** 1 Department of Psychological Medicine, Institute of Psychiatry, Psychology & Neuroscience, King’s College London, London SE5 8AF, UK; 2 South London & Maudsley NHS Foundation Trust, Maudsley Hospital, Denmark Hill, London SE5 8AZ, UK

**Keywords:** acetazolamide, carbonic anhydrase inhibitor, bipolar disorder, scoping review

## Abstract

Acetazolamide, a carbonic anhydrase inhibitor, is used to treat a variety of ailments. It has been highlighted for its potential to benefit people with bipolar disorders, for whom there are clear current unmet treatment needs. This scoping review sought to synthesise all available evidence related to the potential effects of acetazolamide on symptoms related to bipolar disorder, acceptability and tolerability, and intervention characteristics (e.g., dose and duration). Following publication of the review protocol, the Pubmed, Embase, and PsycInfo databases were searched (all dated to 31 August 2022). A systematic approach was undertaken to identify eligible articles and extract relevant data from these. Five studies were included, assessing a total of 50 patients treated with acetazolamide. Most patients were from two open-label trials, while the others were case reports. Approximately one third of patients were experiencing psychosis or mania before treatment initiation, and one third had refractory depression. Forty-four percent of patients were estimated to achieve a response (not seemingly affected by the baseline episode type, acetazolamide dose, or duration), while a further 22% appeared to experience minimal benefits from the intervention. Acetazolamide was generally reported to be tolerated well and acceptable for up to 2 years, although reporting for acceptability and tolerability was suboptimal. The reviewed evidence is extremely limited in size and methodology (e.g., no randomised studies, blinding, or standardised outcome assessment). We posit that the current findings are sufficiently encouraging to recommend substantive clinical trials, but we emphasise that at present, the evidence is exceedingly preliminary, and there remains evident uncertainty as to whether acetazolamide could be a viable treatment for bipolar disorders.

## 1. Introduction

Bipolar disorders (BD) are a leading cause of disability globally [1]. Several factors contribute to this, including a high prevalence (~2–4% lifetime) [2], early onset and frequent lifetime episode recurrences [3], as well as the debilitating effects of mania, depression, and functional and cognitive impairments which frequently persist in periods of remission from acute episodes, as well as subsyndromal symptoms between episodes [3]. Burden calculations are also likely underestimated due to the high rates of undiagnosed bipolar disorders [4]. Notwithstanding the numerous effective treatments for people with BD, these frequently confer a variety of challenges, ranging from risk of affective switch (to symptoms or episodes of the opposite pole), tolerability, and contraindication due to comorbidities or other medication interactions to variable interindividual clinical effectiveness [5]. Some examples here are the safety profile of first-generation antipsychotics (FGAs) as well as negative symptoms (e.g., flattened affect or avolition) and tolerability issues in second-generation antipsychotics (SGAs) (e.g., metabolic) as well as FGAs and the unsuitability of valproate medications for women with childbearing potential, the need for monitoring to ensure non-toxicity with lithium, pharmacokinetic interactions with carbamazepine, risk of serious rashes with lamotrigine, and a manic switch with antidepressants [6,7,8]. Treating bipolar depression is a particular challenge [6,7,8]. Only a small number of medications available for BD are effective against both manic and depressive symptoms as well as for maintenance therapy, and many patients require combination treatments to achieve sufficient symptom control [6,7,8]. Many still experience rapid cycling bipolar disorders, for whom the evidence base for treatments is limited [6]. These challenges with current treatments are acknowledged by well-regarded, widely used clinical guidelines [7,8] and are supported by up-to-date systematic reviews and meta-analyses (e.g., those focused on acute treatment of mania [9], depression [10], maintenance treatment [11], and rapid cycling [6]). Each of the referenced syntheses here states a need for increased clinical trials, both for existing and new interventions.

Acetazolamide is a carbonic anhydrase inhibitor which increases GABAergic transmission and is used widely for a range of indications [12]. It is currently licensed in the UK for glaucoma, epilepsy, and abnormal fluid retention [13]. Since its first use as a diuretic ~70 years ago, acetazolamide was first indicated for its putative value as a psychotropic almost 40 years ago, showing acute and prophylactic antipsychotic effects, particularly in patients with psychosis [14]. Focus on acetazolamide later shifted to affective presentations, as many anticonvulsant medications were found to be effective BD treatments [15]. Since GABA is decreased during both depression and mania, anticonvulsants and GABA agonists can have mood-stabilizing effects, especially in light of the neurotransmission effects which have been implicated in affective episodes [12,15]. However, there have not been, to our knowledge, any robust randomised controlled trials or recent calls for examining this intervention.

## 2. Aims

This scoping review protocol aims to identify and synthesise the available evidence relating to acetazolamide’s potential to be examined as a putative intervention for people with bipolar disorders. Because the literature to date is scant, the scope of the review was kept broad, and we included studies not constrained by their methodological design or outcomes reported. The following research questions were pre-specified prior to undertaking this review:

1. Are there indications that acetazolamide can be effective for people with BD in terms of core affective symptomatology (mania, depression, and mixed affective states)?
2. What are the putative effects of acetazolamide on broader important outcomes such as psychosocial and cognitive functioning and quality of life?
3. Are there indications that acetazolamide could be beneficial prognostically (i.e., in reducing relapse)?
4. How acceptable might acetazolamide be for patients to take in the short or long term? This is considered in terms of adherence and continuation of the medication over time in combination with reports of tolerability and safety. We note that long-term use is generally cautioned with this intervention.
5. Are there indications of what might constitute a therapeutic dose or duration of acetazolamide for BD?

## 3. Methods

### 3.1. Protocol and Registration

We conducted this scoping review in accordance with the Preferred Reporting Items for Systematic Reviews and Meta-Analyses Extension for Scoping Reviews (PRISMA-ScR) guidelines [16] (see Appendix A). The scoping review protocol was published before the systematic search was undertaken (30 August 2022) [17].

### 3.2. Eligibility Criteria

To be included in the scoping review, articles needed to meet three criteria. They had to describe acetazolamide (intervention) for people with bipolar disorders (participants/population) and report an outcome relevant to people with BD (outcomes). The studies could be of any design (including preclinical, if meeting the criteria specified above) and could include any comparator intervention or control or a lack thereof. These criteria are purposefully inclusive, seeing as the evidence base for this intervention/population is limited, and in order to establish acetazolamide’s potential as an intervention for BD, it was deemed important to consider all relevant evidence at this stage. We were therefore also inclusive in terms of publication date (any), publication status (including non-peer reviewed articles), and article language (where a translation was feasible). Thus, the only exclusion criterion was a lack of reporting on clinical outcomes of acetazolamide for people with bipolar disorders.

### 3.3. Information Sources and Search Procedure

**Figure 1:**
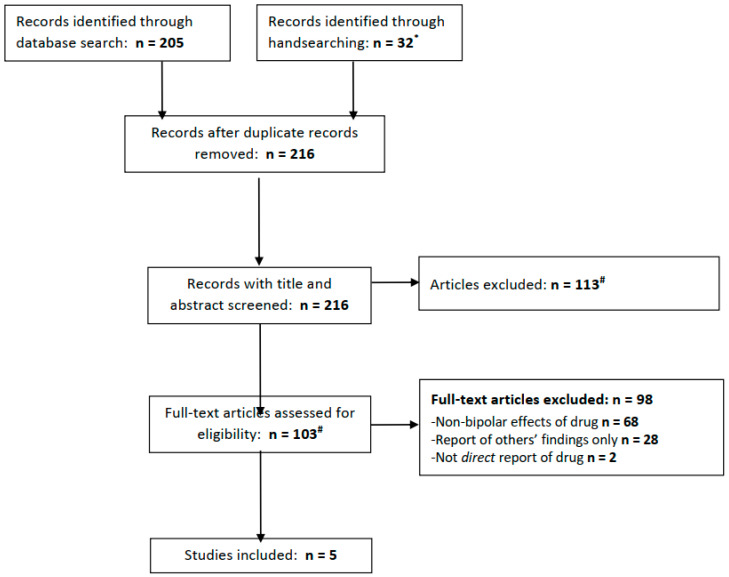
PRISMA flow diagram for included studies. * We searched clinicaltrials.gov for acetazolamide + bipolar (0 results), mania (0 results), depression (0 results), or more broadly, psychiatric disorder (7 results, of which 1 is ongoing for schizophrenia, 4 excluded people with psychiatric diagnoses and 2 did not mention relevant population or outcomes.) # Three full texts not found from any source.

The PubMed, Embase, and PsycInfo databases were searched for articles available from inception to 31 August 2022. The terms used to search these databases were as follows (in all fields): ((Acetazolamide) or (Diamox)) AND ((bipolar) or (mania)). In attempting to identify any articles not retrieved from these databases and highlight the relevant grey literature, clinicaltrials.gov was searched using the same terms, and all articles eligible for a full-text review (see below) were handsearched for possibly relevant additional references. Any articles identified in handsearching were added to the search results (Figure 1).

### 3.4. Selection of Sources of Evidence

Each record retrieved from the database search was identified concurrently. The initial deduplication of records was undertaken in Ovid. All remaining records were imported into Rayyan open-source review management software [18], and a second deduplication check was run. Rayyan software was used to record the review status of each retrieved article. All articles were subject to title and abstract screening by one author (R.S. or S.O.). Any article appearing relevant to the review topic was subject to a full-text review by the same author. The full text review was undertaken to ascertain its eligibility for inclusion using our pre-specified criteria. Subsequently, the two reviewers attained consensus on eligible studies with the support of an additional reviewer (A.H.Y.) where any uncertainty remained. This process was also followed for additional articles identified during handsearching.

### 3.5. Data Charting and Items

All included articles were subsequently scoured to extract relevant data using a standardised form (MS Excel) including a range of pre-specified variables of interest. This data extraction was conducted by one reviewer and checked by another (R.S., N.Y., and S.O.). Any discrepancies were resolved by consensus between the three review authors. For any articles not written in English, the initial search and data extraction were conducted using internet-based software (Google Translate) and were subsequently checked by a speaker of that language to professional proficiency. The pre-specified variables extracted from each included article pertained to the following: bibliographic information about the study or authors (reference, location, and trial registration); study design (design factors, setting, blinding, and follow-up); population (diagnosis, eligibility and assessment, current symptoms, and comorbidities); interventions (continuation therapies, concomitant treatments, comparison groups, dose, and treatment duration); participant characteristics (number, gender, and age); intervention effects (BD-relevant symptoms or clinical effects (see below), tolerability, adherence, and discontinuation); methodological considerations; and other notable features of the study. The primary outcome was any measure(s) pertaining to the core symptoms of bipolar disorders (depression or mania), with regard to improvement (either as a continuous score or proportion of response/remission where applicable) or worsening (either as a continuous score or proportion of relapse, where applicable). Secondary outcomes related to BD clinical outcomes included broader assessments of functioning or other related symptoms (e.g., psychosis or anxiety).

### 3.6. Synthesis of Results

A formal critical appraisal of study risk of bias or quality was not undertaken, as is standard for scoping reviews, particularly when studies vary in design, participants, and outcomes. A narrative synthesis was planned to summarise the extracted data and observe emerging patterns. Ultimately, the synthesis of results was pre-decided to be categorised as follows, with the overarching objective being to ascertain the potential for acetazolamide to be considered in future clinical trials as a putative intervention to help people with bipolar disorders:

6. Quantity of data available to date;
7. Evidence of benefits (i.e., primary and secondary outcomes specified above);
8. Evidence of drawbacks (e.g., non-adherence, tolerability issues, and discontinuation);
9. Intervention-specific considerations, including dose and duration of treatment;
10. Methodological considerations, including type and strength of evidence and potential effect modification.

## 4. Results

### 4.1. Selection of Studies

In total, the electronic database searches yielded 205 records (PubMed 15; Embase 110; and PsycInfo 80), of which 32 were retrieved from handsearching, including 7 results from a search of clinicaltrials.gov (of which none were relevant; see Figure 1). After removing duplicate records, 216 articles remained and were subject to screening. Screening led to the exclusion of 113 articles, leaving 103 whose full texts were reviewed. The majority of the articles were excluded at this stage, leaving five which met our eligibility criteria.

### 4.2. Characteristics of Included Studies

Two studies were single-arm, open-label trials, treating 16 [15] and 30 [19] patients with acetazolamide, respectively. The larger study included patients with “atypical psychoses” (with eligibility as per clinical judgement) who all had manic-depressive features but may not have met the modern diagnostic criteria for bipolar disorders [19]. The smaller open-label study instead recruited patients with primary bipolar disorders according to DSM-III [15]. The other three studies were case reports, either including a single case [20], two cases [21], or multiple cases where only one was treated with acetazolamide and was therefore included in this review [22]. The latter article was written in Japanese. Between the studies, the (average) age ranged from 13 to 49. Only one study reported specific participant exclusion criteria [15]. No studies specifically reported the clinical setting in which treatment took place. No patients, clinicians, or outcome assessors were blinded to the intervention. Table 1 contains further details regarding the included studies’ characteristics, while Table 2 contains details of the included studies’ intervention characteristics.

### 4.3. Quantity of Data Available

When incorporating the 5 studies, a total of 50 participants were treated with acetazolamide. Most were female (86%). There was wide variety in terms of the state at baseline. Summarising the studies from largest to smallest, the participants were in different phases (although primarily psychotic; *n* = 30) [19] or had refractory depression (or rapid cycling; *n* = 16) [15], psychotic mania (*n* = 1) or depression (*n* = 1) [21], refractory depression (*n* = 1) [22], or mania (*n* = 1) [20]. All aforementioned states were defined via clinical judgement.

### 4.4. Evidence of Benefits (i.e., Primary and Secondary Outcomes Specified Above)

Of the 50 patients, there was an approximately 44% substantive response rate, with a further 22% appearing to have a partial or possible improvement and the remaining 34% being non-responders.

When separately calculating the responses for those who were previously presenting with psychosis or mania (*n* = 32) and (usually refractory) depression (*n* = 18), the rate of response was comparable (both 44%), with slightly more depressed patients showing signs of a definitive non-response (50%) than mania or psychosis (36%).

The largest open-label study (bipolar psychoses) reported some benefits to 73% of patients, although for 33% of these were categorised (according to clinical judgement) as “slightly effective”, which the current authorship judged to likely not meet the usual criteria for a binary definition of response (i.e., response likely categorised for 40%) [19].

The other open-label study (refractory depression) defined the responses according to the Global Assessment of Functioning (GAF), and 44% were reported to achieve a response which was sustained (reportedly with no loss or gain in magnitude during long-term treatment). In the patients continuing to take acetazolamide, the overall psychiatric symptom scores (Brief Psychiatric Rating Scale, although weighted highly for psychotic symptoms) indicated a reduction in severity from moderately ill (baseline mean of 41) to subthreshold (scores at each following averaging of 24–27) (severity thresholds according to Leucht et al., 2005 [23]) [15].

The case report of two patients (one with psychotic mania and one with depression at initiation) revealed a sustained response in both, with continued remission while taking acetazolamide according to clinical judgement [21].

In the mania case report, the participant reduced in severity from having a Bech-Rafaelson Mania Scale score of 19 (mild-to-moderate mania) to 8 (remission) (severity thresholds according to Bech, 2002 [24]) [20].

Finally, in the case report of one patient with refractory depression, no response was observed in the first 6.5 months when concomitantly taking acetazolamide with carbamazepine and then valproate. After initiation of lamotrigine alongside a dose reduction of acetazolamide (from 875 to 500 mg), minimal improvement was observed at 4 and 8 weeks, followed by a significant amelioration of symptoms (in addition to functioning) by 28 weeks. Despite the acetazolamide dose reduction prior to the response, the improvement was attributed to lamotrigine (the article’s focus). It was also not explicit that acetazolamide was continued for the full 28 week follow-up, and this case was thus categorised under the “partial response” category above [22].

### 4.5. Evidence of Drawbacks

Data reporting for discontinuation was scant. In the larger open-label study, it was noted that discontinuation usually occurred upon remission [19]. Non-responders in the other open-label study discontinued after 6 weeks, while all responders continued for more than 6 months (with 6 for up to 12 months, 3 for up to 18 months, and 2 for 2 years) [15]. Of the case reports, one did not report discontinuation, and one took acetazolamide for 54 weeks (approximately 7 months before response and 5 months afterwards) [22], while this was not reported for one further responder and one responder discontinued after one year at the request of their family [21]. No other reasons for discontinuation were described. Two studies did not report tolerability data [15,22]. The other three studies reported generally good tolerability, two of which also reported no concerns from blood safety markers. In terms of patient-reported events, one described a sedation effect and numbness in the fingers [19], while case reports described transient sluggishness and polydipsia [21], as well as transient nausea, mild sedation, and polyuria [20].

### 4.6. Intervention-Specific Considerations, Including Dose and Duration of Treatment

Concomitant medication: Patients were concomitantly treated with a variety of common treatments at the time of study (1980–1998). While these varied within the studies, the smaller open-label trial included all patients taking thyroxine [15], and individual cases were treated with either valproate plus perazine (which notably were only initiated 3 days before acetazolamide) [20], carbamazepine later switched to valproate and then lamotrigine [22], or chlorpromazine (whose dose was increased at the same time as acetazolamide initiation but was later discontinued) [21], with one taking no other medication [21].

Duration: The shortest acetazolamide exposure was 17 days (mania responder, although the patient was concomitantly treated with other antimanic agents which were only initiated three days prior and had been unmedicated before that) [20]. Other cases were treated for approximately one year (one late or possible responder [22] and two responders [21]). Of the two open-label trials, one treated patients for 6 weeks but responders for up to 2 years [15], and the other did not report their duration of exposure but did follow up with patients for 2 years [19]. This suggests that while the duration of effectiveness is uncertain, it is possible to undergo long-term treatment with this intervention.

Dose: Multiple studies titrated patients from 250 mg/day (a dose which did not appear effective). One report of two cases reported the effectiveness of 400–500 mg [21], and the two open-label studies (plus one case report) employed a maximum tolerated dose of 1000 mg with effectiveness reported overall [15,19,20].

### 4.7. Methodological Considerations, Including Type and Strength of Evidence and Potential Effect Modification

It is important to emphasise that despite synthesising these data from participants across studies (above), there was variation in the patient characteristics, response assessment (including clinical judgement vs. validated assessments), study design (with no randomised or blinded trials), and intervention characteristics. When comparing responders and non-responders across (and within) studies, there did not appear to be a clear pattern of effect modification by dose (500–1000 mg across studies), duration (from 17 days to 2 years in responders and from 6 weeks to 2 years in non-responders), concomitant treatment (although only 1 patient (responder) was on monotherapeutic acetazolamide), or baseline state.

## 5. Discussion

We identified treatment of 50 people with bipolar disorder with acetazolamide. Of the 50 treated patients, almost two thirds were experiencing psychosis or mania, and one third had depression. Most of the latter patients were categorised as refractory prior to acetazolamide treatment, and the response rates (44%) appeared to be equivalent across both poles. Although an overall response rate of 44% appears to be relatively low, it is not far from the response rates reported after other medications, particularly for depression. For example, several randomised trials of currently recommended medications have achieved similar rates of response as monotherapies [25]. As noted earlier, few medications recommended for bipolar disorder are effective against both the mania and depression poles.

It is worth noting here that topiramate, which is also a weak carbonic anhydrase inhibitor, initially showed signs of potential for treating people with bipolar disorder, and this has since been largely classed as ineffective. Early evidence of topiramate suggested effectiveness in around 35% of patients (although the cited trial was in young people) [26], reducing by around 10% in larger trials [27] and similar to the placebo response in both. It is possible that the story would be similar if examining acetazolamide intensively for its potential effectiveness in patients with bipolar disorder. However, we consider that the equivalent response rates we report here in people with refractory depression, and the high prevalence of treatment-resistant depression in bipolar disorders [28], warrant rigorous investigation. The lack of randomised controlled trials or, to our knowledge, any interventional study in the last 25 years is surprising in this respect. However, it may be explained by the introduction of new substances in the 1990s that held promise for the treatment of bipolar disorders, including new antiepileptics and second-generation antipsychotics as well as the shift of research trials towards investigating these.

Below we contextualise the current findings alongside previous indications pertaining to the review’s aims, as stated in the introduction to this article:

(1) Are there indications that acetazolamide can be effective for people with BD in terms of core affective symptomatology (mania, depression, or mixed affective states)?
(2) The current findings suggest that there are indications that acetazolamide can be effective for BD in terms of mania [19,20,21] and depression [15,21], although we have not identified any evidence supporting (or contravening) its use for mixed episodes.
(3) What are the putative effects of acetazolamide on broader important outcomes, such as psychosocial and cognitive functioning and quality of life?
(4) Most included studies only assessed core bipolar disorder symptoms (and mostly according to clinical judgement, which therefore may have included the level of psychosocial functioning or global recovery), although one study identified positive effects on global functioning [15], and another specified its inclusion in the definition of response (although the latter study, for one patient, indicated the weakest effect of the intervention).
(5) Are there indications that acetazolamide could be beneficial prognostically (i.e., in reducing relapse)?
(6) Although all included studies examined patients who were symptomatic at the time of acetazolamide initiation, four out of five reported long-term treatment (usually over one year) and suggested that its effects were maintained over time. Some specifically reported a lack of relapse after response [15,21], which supports its potential for maintenance trials.
(7) How acceptable might acetazolamide be for patients to take in the short or long term?
(8) Despite limited reporting, no studies reported serious adverse events, and discontinuation in the short term was relatively infrequent. While these data are preliminary, the side effects reported are similar in nature and severity to many other medications recommended for common and disabling physical and mental health conditions, including bipolar disorders. Because many patients were treated for more than one year, this increases confidence somewhat in the acceptability of intervention in both the acute and maintenance phases. However, the other literature has warned of the safety of acetazolamide in people with existing renal, hepatic, or pulmonary problems [29,30,31].
(9) Are there indications of what might constitute a therapeutic dose and duration of acetazolamide for BD?
(10) Because most studies examined the long-term use of acetazolamide at 500–1000 mg, these would seem to be adequate for future rigorous trials. It is notable that many studies did not suggest that higher doses are more effective, and therefore a regime where a dose is increased from 500 mg up to 1000 mg may be sensible where the response is not optimal and where this can be tolerated.

### Clinical Possibilities

Acetazolamide’s potential for people with bipolar disorder may be reinforced by its potential for rare illnesses with similar manifestations to bipolar disorder, including CADASIL [32] and Kleine-Levin syndrome [33]. It has also been proposed as a potential intervention for Alzheimer’s disease (AD), which shares some similarities with bipolar disorder in cognitive impairment, other potential overlapping interventions (e.g., lithium), and risk for AD in people with bipolar disorders [34].

In addition to topiramate (mentioned above), another anticonvulsant with carbonic anhydrase inhibitory properties (albeit weaker than acetazolamide), zonisamide, has similarly been suggested for its potential benefit in mania [35] and depression [36], although its tolerability has been questioned [37].

Acetazolamide has been trialled for its potential to overcome the side effects of other (relevant) medications, including weight gain [38] and extrapyramidal symptoms (alongside thiamine [39]). It has also been suggested for potential value in treating lithium-induced nephrogenic diabetes insipidus (Li-NDI), with some inconsistent reports claiming effects on reducing polyuria or attenuating the biological severity of illness [40,41,42], although cautions have been raised over its efficacy and safety (particularly regarding its effects on eGFR) as well as its reductive effect on serum lithium levels [30].

## 6. Strengths and Limitations

While we believe the strength of this work is in synthesising all relevant evidence pertaining to acetazolamide’s potential for benefitting those with bipolar disorders, thus potentially reigniting a dormant research pathway, there are related weaknesses. First, we have included evidence focused on a variety of bipolar-type affective psychoses, but not every single patient of the 50 may have had a de facto bipolar disorder. Conversely, we excluded evidence related to non-affective psychoses, although these have been a driving force for building evidence bases for other bipolar medications. One example of an excluded study here reported on the benefits for people with schizophrenia, but it was concluded that this may be a treatment with potential for a variety of chronic mental illnesses, including bipolar disorders [14]. Because the reviewed evidence base is more than 25 years old, it is composed mainly of case reports. The articles have not been published in high-impact journals, and there were even some articles identified from database and handsearching which the authors were not able to locate, despite extensive searching by the authors and institutional libraries. We describe these here. One from 1973 appears to be a double blind study on carbonic anhydrase inhibitors for prophylaxis and mania [43]. Another published five years later by the same authors specifically names acetazolamide as the focus of a mania trial [44]. Finally, there appears to be a case study of an unspecified carbonic anhydrase inhibitor for atypical psychosis from the same authors as two of our included studies [45]. The a priori decision to include case reports enabled more than two studies to be included in our review (increasing our sample size by four patients), but this clearly increased methodological heterogeneity, which was already extensive. It also raised an issue of publication bias, since positive case reports of undemonstrated interventions are more likely to be published than negative reports. We finally emphasise the paucity of evidence and resultant stark uncertainty from the literature. In particular, we note the absence of evidence for mixed affective episodes and the lack of focus on the overall course of bipolar disorder in the longer term after acetazolamide administration. Other points included earlier in the discussion should also be considered alongside these limitations (i.e., non-use of standardised or validated outcome assessments and scarce examination of global functional recovery, safety, and dose and duration effects). Moreover, the included studies were—as discussed—heterogeneous in terms of their design, participants, intervention characteristics, and outcomes assessed. This heterogeneity limited our ability to compare their results, adding to the uncertainty and inability to draw conclusions.

Despite this concerning uncertainty, we summarise that this preliminary evidence indicates that acetazolamide is potentially effective in the treatment of both mania and depression and as maintenance therapy in bipolar disorders. Randomised controlled trials are warranted to investigate its short- and long-term effects in the treatment and prevention of manic, depressive, and mixed feature episodes.

## Figures and Tables

**Table 1:** Characteristics of included studies.

Reference	Study Design	Study Population	Episode Type	Mean Age	Gender (F/M)	Diagnostic Criteria	Continuation or Concomitant Treatments
Brandt et al., 1998 [20], Germany	Case report	Bipolar Disorder (*n* = 1)	Manic	39	0/1	NR	Valproate (serum level 17.6 μg/mL) + Perazine, both initiated 3 days before AZ.
Fukuma & Inoue, 1980 [21], Japan	Case series	Periodic Atypical Hypomania (*n* = 2)	Manic (*n* = 1) Depressive (*n* = 1)	13	2/0	NA	Mania case: Chlorpromazine (from 60 mg; dose increased to 110 mg at AZ initiation; later discontinued). Depression case: medication-free.
Fogelson & Sternbach, 1997 [22], USA	Case report	Bipolar Disorder Type I (*n* = 1)	Refractory rapid cycling, currently depressive	33	1/0	DSM IV	AZ initiated with carbamazepine (1500 mg; discontinued after 4.5 months), valproate (750 mg; from month ~4 to 6 of AZ), and then lamotrigine (200 mg; from month ~6 to 12 of AZ.)
Hayes et al., 1994 [15], USA	1-arm open-label	Bipolar Disorder (*n* = 12) Schizoaffective Disorder (*n* = 4)	Refractory depressive or rapid cycling	48.5	13/3	DSM III R	Thyroxine (physiological doses maintained throughout) + treatment as usual (mood stabilisers and/or ECT *).
Inoue et al., 1984 [19], Japan	1-arm open-label	Puberal Periodic Psychosis (*n* = 6) Presenile Atypical Psychosis (*n* = 7) Atypical Psychosis (*n* = 8) Atypical Manic-Depressive Psychosis (*n* = 2) Atypical Schizophrenia (*n* = 7)	Varied	35.5	27/3	NA	Treatment as usual (low-dose antipsychotics in many cases, with doses decreasing as symptoms stabilised).

Abbreviations: AZ = acetazolamide. * Responders: 2 monotherapy valproate; others were combinations including lithium, carbamazepine, phenytoin, and a neuroleptic. Non-responders: 1 monotherapy lithium; others were combinations as described above.

**Table 2:** Characteristics of acetazolamide treatment.

Reference	Dose	Duration	Follow-Up	Main Outcomes	Additional Outcomes	Tolerability
Brandt et al., 1998 [20], Germany	1000 mg/day	17 days	No	BRMAS score reduced from 19 to 8.	Influence of concomitant treatments: low valproate dose; perazine is not known as a potent antimanic.	Good; transient nausea, mild sedation, polyuria.
Fukuma and Inoue, 1980 [21], Japan	250–500 mg/day	52 weeks	1 case, duration NR	Clinical judgement: 1 case sustained remission, 1 case no relapse during treatment.	1 case discontinued after 8 months, immediate relapse. Authors concluded effect requires >2 weeks at >400 mg.	Transient sluggishness and thirst.
Fogelson and Sternbach, 1997 [22], USA	500–875 mg/day	54 weeks	No	Clinical judgement: no response at 750–875 mg/day with carbamazepine or valproate. Moderate symptom improvement at 500 mg/day with lamotrigine.	Patient non-response to various previous medications. No pre-acetazolamide data, so unclear if outcomes attributable to acetazolamide.	NR.
Hayes et al., 1994 [15], USA	1000 mg/day	6 weeks	2 years	44% response rate. In responders: BPRS scores decreased from 41 to 24–27; GAF scores increased from 49 to 76–85.	Responders: 6 BD, 1 SAD Non-responders: 6 BD, 3 SAD No efficacy changes at follow-up.	NR.
Inoue et al., 1984 [19], Japan	500–1000 mg/day	NR	2 years	Clinical judgement: markedly effective: 23%, effective 17%, slightly effective 33%, ineffective 27%.	Antipsychotic effect in 60%. Prophylactic effect in 30% (including 10/13 non-responders to lithium or carbamazepine).	Sedation, numbness in fingers. No effects on serum electrolytes, cardiac function, liver or renal function.

Abbreviations: BD: bipolar disorder, BPRS: Brief Psychiatric Rating Scale, BRMAS: Bech-Rafaelsen Mania Scale, GAF: Global Assessment of Functioning, NR: not reported, USA: United States of America, and SAD: schizoaffective disorder.

## Data Availability

Please make any requests to the corresponding author.

## Appendix A

Preferred Reporting Items for Systematic Reviews and Meta-Analyses extension for Scoping Reviews (PRISMA-ScR) checklist.

**Section**	**Item**	**PRISMA-ScR Checklist Item**	**Reported on Page No.**
TITLE			
Title	1	Identify the report as a scoping review.	1
ABSTRACT			
Structured summary	2	Provide a structured summary that includes (as applicable) background, objectives, eligibility criteria, sources of evidence, charting methods, results, and conclusions that relate to the review questions and objectives.	1
INTRODUCTION			
Rationale	3	Describe the rationale for the review in the context of what is already known. Explain why the review questions or objectives lend themselves to a scoping review approach.	1–2
Objectives	4	Provide an explicit statement of the questions and objectives being addressed with reference to their key elements (e.g., population or participants, concepts, and context) or other relevant key elements used to conceptualise the review questions or objectives.	2
METHODS			
Protocol and registration	5	Indicate whether a review protocol exists, state if and where it can be accessed (e.g., a web address), and if available, provide registration information, including the registration number.	2
Eligibility criteria	6	Specify characteristics of the sources of evidence used as eligibility criteria (e.g., years considered, language, and publication status), and provide a rationale.	2–3
Information sources *	7	Describe all information sources in the search (e.g., databases with dates of coverage and contact with authors to identify additional sources), as well as the date the most recent search was executed.	3
Search	8	Present the full electronic search strategy for at least 1 database, including any limits used, so that it could be repeated.	3
Selection of sources of evidence ^†^	9	State the process for selecting sources of evidence (i.e., screening and eligibility) included in the scoping review.	3
Data charting process ^‡^	10	Describe the methods of charting data from the included sources of evidence (e.g., calibrated forms or forms that have been tested by the team before their use and whether data charting was performed independently or in duplicate) and any processes for obtaining and confirming data from investigators.	3
Data items	11	List and define all variables for which data were sought and any assumptions and simplifications made.	3
Critical appraisal of individual sources of evidence ^§^	12	If performed, provide a rationale for conducting a critical appraisal of included sources of evidence and describe the methods used and how this information was used in any data synthesis (if appropriate).	3
Synthesis of results	13	Describe the methods of handling and summarizing the data that were charted.	3
RESULTS			
Selection of sources of evidence	14	Give numbers of sources of evidence screened, assessed for eligibility, and included in the review, with reasons for exclusions at each stage, ideally using a flow diagram.	4
Characteristics of sources of evidence	15	For each source of evidence, present characteristics for which data were charted and provide the citations.	4–5
Critical appraisal within sources of evidence	16	If performed, present data on critical appraisal of included sources of evidence (see item 12).	4–5
Results of individual sources of evidence	17	For each included source of evidence, present the relevant data that were charted which relate to the review questions and objectives.	4–5
Synthesis of results	18	Summarise or present the charting results as they relate to the review questions and objectives.	4–5
DISCUSSION			
Summary of evidence	19	Summarise the main results (including an overview of concepts, themes, and types of evidence available), link to the review questions and objectives, and consider the relevance to key groups.	6–7
Limitations	20	Discuss the limitations of the scoping review process.	7
Conclusions	21	Provide a general interpretation of the results with respect to the review questions and objectives, as well as potential implications or next steps.	6–7
FUNDING			
Funding	22	Describe sources of funding for the included sources of evidence, as well as sources of funding for the scoping review. Describe the role of the funders of the scoping review.	8
JBI = Joanna Briggs Institute; PRISMA-ScR = Preferred Reporting Items for Systematic Reviews and Meta-Analyses Extension for Scoping Reviews. * Where sources of evidence (see second footnote) are compiled from, such as bibliographic databases, social media platforms, and web sites. ^†^ A more inclusive or heterogeneous term used to account for the different types of evidence or data sources (e.g., quantitative or qualitative research, expert opinion, and policy documents) that may be eligible in a scoping review as opposed to only studies. This is not to be confused with *information sources* (see first footnote). ^‡^ The frameworks by Arksey and O’Malley (6) and Levac et al. (7) as well as the JBI guidance (4 and 5) refer to the process of data extraction in a scoping review as data charting. ^§^ The process of systematically examining research evidence to assess its validity, results, and relevance before using it to inform a decision. This term is used for items 12 and 19 instead of “risk of bias” (which is more applicable to systematic reviews of interventions) to include and acknowledge the various sources of evidence that may be used in a scoping review (e.g., quantitative or qualitative research, expert opinion, and policy documents). *From:* Tricco AC, Lillie E, Zarin W, O’Brien KK, Colquhoun H, Levac D, et al. PRISMA Extension for Scoping Reviews (PRISMAScR): Checklist and Explanation. Ann Intern Med. 2018;169:467–473. doi: 10.7326/M18-0850.			
